# Are They Buying It? United States Consumers’ Changing Attitudes toward More Humanely Raised Meat, Eggs, and Dairy

**DOI:** 10.3390/ani8080128

**Published:** 2018-07-25

**Authors:** C. Victor Spain, Daisy Freund, Heather Mohan-Gibbons, Robert G. Meadow, Laurie Beacham

**Affiliations:** 1 American Society for the Prevention of Cruelty to Animals (ASPCA), New York, NY 10018, USA; Daisy.Freund@aspca.org (D.F.); Heather.Mohan-Gibbons@aspca.org (H.M.-G.); lauriemara@gmail.com (L.B.); 2 Lake Research Partner, Washington, DC 20036, USA; bmeadow@lakeresearch.com

**Keywords:** farm animal welfare, chicken, dairy, eggs, beef, consumer confusion, willingness to pay, welfare certification, humane meat, food labels

## Abstract

**Simple Summary:**

The lack of a consistent approval process for animal welfare claims in the US has allowed for misleading labeling of meat, eggs, and dairy. Products that do have meaningful welfare certifications tend to be more expensive. We administered a survey to determine consumers’ attitudes towards welfare certifications and the willingness to pay for foods from animals raised under more humane conditions. Most respondents (78%) thought it was important to know that animal-welfare assessments are conducted by an independent third party or the federal government (and not only the industry producer). The majority of respondents would be willing to pay extra for foods with a trustworthy welfare certification both in supermarkets and in restaurants. Our findings underscore the importance of eliminating fraudulent or misleading claims that can erode consumer trust and suggest that retailers can best serve consumers who are interested in higher welfare products by stocking products with certifications that convey meaningful information on the animal welfare standards from the source farms.

**Abstract:**

This survey research sampled 1000 US (United States) consumers of meat, eggs, and dairy on their attitudes towards the welfare of farm animals and the willingness to pay for products with trustworthy welfare certifications. Most respondents (70%) reported paying attention to labels that indicate how the animals were raised and 78% believed there should be an objective third party to ensure farm animal welfare. The weighted average of the marginal willingness to pay for products raised under a trustworthy welfare certification was $0.79 for eggs (a 32% premium) and $0.96 for 1 lb. of chicken breast (a 48% premium). In addition, 57% of respondents reported they would be likely to choose a restaurant because it serves welfare-certified animal products and are also willing to pay ≥$5.00 extra per entrée. These findings suggest that many US consumers, particularly millennials, would be willing to seek out higher welfare products if they trust the label claims.

## 1. Introduction

Since World War II, food production in the US (United States) has become increasingly industrialized. This trend had the effect of creating an abundant supply of inexpensive food in part by consolidating small farms into integrated agriculture businesses that compete through efficiency and strive to produce more with a smaller input of resources or labor [1,2,3]. An increasingly urban population today has almost no access to farms and often lacks an understanding of the scale and common practices of modern facilities [2,4]. This distance between consumers and farms and the primacy of efficiency has contributed to a shift in the meat, egg, and dairy industries from extensive, pasture-based, practices to efficiency-focused, intensive, and indoor-based systems [5].

While there has been rising public concern over these and other practices from ethical, public health, and environmental perspectives [6], there are few effective checks on how producers raise farm animals [5,7]. Animals raised for food are among the least-protected class of animals under US law. There are no federal laws protecting or dictating animal treatment on farms and the majority of US states expressly exempt farm animals or certain standard farming practices from their anti-cruelty provisions. Only two federal laws cover farm animals during transport and slaughter, which includes The Humane Methods of Slaughter Act of 1958 and the Twenty-Eight Hour Law enacted in 1994 [8,9]. Both exempt all poultry species, which make up 98% of land animals raised for food [10,11]. Farm animals are explicitly excluded from protection under the Animal Welfare Act [12]. In response to the lack of federal oversight of farm animal welfare, 11 states have recently passed laws banning cage confinement of egg-laying hens, mother sows, and/or veal calves [13].

The large majority of meat, eggs, and dairy products (i.e., animal source foods (ASF)) in the US come from animals raised under conditions set by their respective industries. Of the estimated 371 million laying hens in the US in 2017, only 42 million (11.5%) are considered cage-free [14]. The remainder are raised indoors in conventional cages sometimes known as battery cages. The US National Chicken Council whose member corporations represent approximately 95% of broiler chickens raised (i.e., young chickens raised for meat) reports that these animals are all raised entirely indoors in “growout houses” with no outdoor access [15,16]. In the US, the predominant housing system for pregnant sows is gestation stalls in which the sows are confined without the ability to turn around [17]. There are a number of higher welfare certification programs in the US with varying standards. Only three programs, Certified Humane, Global Animal Partnership, and Animal Welfare Approved, operate nationwide and require 100% compliance with standards that require, at minimum, enriched, spacious, cage-free environments verified by independent auditors on farms [18,19,20,21]. The number of animals raised under these certifications as of 2017 is approximately 500 million (i.e., approximately 5% of animals raised for ASF in the US) [19,22]. The details of the standards for these certifications vary, but all ban caging, crating, crowding, and require enrichment necessary for animals to perform natural behaviors. Use of antibiotics for growth promotion is banned under these certifications, but ill animals must be treated with therapeutic antibiotics. These certification programs also have standards for transport and slaughter, which has been a priority for species not covered by US laws.

Several previous studies have concluded that consumers in North America and Europe place significant value on food carrying apparent assurances of higher welfare practices [23,24,25]. However, the current approval process for animal welfare claims leads to misleading labeling of meat, eggs, and dairy. A report by the Animal Welfare Institute showed that the USDA (United States Department of Agriculture, Washington, DC, USA) often requires no substantiation of animal rearing claims and, when it does, the evidence farms must submit is inadequate and far below consumer expectation [26]. Some label designations such as “natural” have no impact on animal welfare and may be diluting the demand for higher welfare products by offering consumers false security. As an example of the level of misunderstanding, a 2015 Consumer Reports study found that consumers believed that a natural label indicates that animals went outdoors when there are no such requirements for this label [27]. Similarly, consumers demonstrated the willingness to pay for many types of meat labeled “no added hormones” even though this is a meaningless term when it appears on pork, eggs, chicken, or turkey since law already prohibits the use of hormones on birds and swine [28,29]. The term “humane” is undefined by law and is, therefore, entirely subjective [30].

Awareness and skepticism are growing around labels related to animal treatment. Responding to consumer complaints about the lack of clarity around the term “natural”, the US Food and Drug Administration recently sought public comment asking for input on what the word should mean. In 2017, the third largest U.S. chicken company, Sanderson Farms LLC, was sued for falsely advertising products that contain a wide range of unnatural and in some cases prohibited substances as “100% Natural” [31]. Several companies that have used the word “humane” on packaging have been challenged in multiple lawsuits [32]. In two cases, the companies Perdue Farms Inc. (Salisbury, MD, USA) and The Kroger Co. (Cincinnati, OH, USA) settled in exchange for removing the claim [33,34]. In 2017, the USDA put forth revised animal welfare standards for the USDA organic program in response to consumer and animal groups revealing the wide gulf between consumer expectation and the reality of large-scale organic animal farms, but the revised rule was later withdrawn [35].

The financial incentives for producers to invest in products with clear labeling and transparent validation of animal welfare are rising, but if consumers are going to pay more, they need assurances that what they are getting is meaningfully better than a conventional product [36]. When making decisions about which meat, eggs, and dairy products to source and stock, businesses such as supermarkets, restaurants, and food processors might, therefore, be interested in consumer demand for—and willingness to pay for—food that reliably comes from higher welfare sources. In order to better describe the current thinking of US consumers, we administered a survey to determine current attitudes regarding the welfare of farm animals raised for ASF, the influence of information about farm animal treatment on changes in purchasing decisions, the willingness to pay for ASF coming from animals raised under more humane conditions, and how key responses differ by demographic subgroups.

## 2. Materials and Methods

This study is a cross-sectional descriptive survey of US consumers of animal source foods (ASF: meat, eggs, and dairy products). The survey was implemented by Lake Research Partners to members of consumer panels who previously volunteered to participate in market research. Participants were emailed an invitation to complete an internet-based survey. Respondents eligible for the survey were US residents 18 years old and older who indicated that they were the primary or co-primary grocery shopper of the household and purchase at least some ASF for their households. In order to reduce the likelihood of preferentially selecting respondents with positive attitudes towards animal welfare, the survey invitation did not mention animal welfare and instead indicated that the topic was “issues that people have been discussing recently about food, eating, and shopping habits,” and no questions related to animal welfare were provided until after the respondent was determined to be eligible for the study. The survey was fielded in 2016 and closed once 1000 eligible respondents were identified.

The survey aimed to assess the consumers’ current purchasing patterns for ASF, attitudes, awareness, and understanding of farm animal conditions and the labeling of food products, and perceived influences of how new information could affect their purchasing patterns. The survey items covered four broad topic areas including (1) respondent characteristics, (2) attitudes towards farm conditions and the meat, egg, and dairy industries such as the level of concern about welfare of animals raised for food, (3) consumers’ considerations when making purchasing decisions for ASF such as the level of attention paid to labels indicating how the animal was raised, and (4) the willingness to pay or travel more for higher-welfare goods. (See Supplemental Materials for full list of items.) For the questions on their willingness to pay, respondents were provided a base price for conventional products and then five discreet choices that covered the price range for commonly encountered brands of eggs or chicken breast with welfare certifications.

In order to maintain a manageable survey length, respondents in each of four subgroups were randomly assigned to receive only half of the items for two sets of questions including the importance of particular practices for indicating good treatment of farm animals and the importance of various types of information when making purchasing decisions. Those subgroups were identified based on responses to the level of concern and the attention paid to labels. When it did not interfere with understandability, the order of items within each question was randomized to minimize any ordering effect. Respondents were also provided a choice for “cannot answer” for all relevant items.

A weighting algorithm was applied so that the summary data on eligible respondents would be representative of typical consumers of ASF (based on previous market research). The characteristics used for weighting were education, age, sex, race/ethnicity, census region, household income, party identification, marital status, parental status, whether the household has pets, and whether the respondent was the primary shopper. Each respondent was given a score based on their reported information and then either weighted up (if they are underrepresented) or weighted down (if they are overrepresented). For analytic purposes, categories indicating a positive response (e.g., concern, agreement, or trust) were combined to create a dichotomous outcome. Responses were summarized as proportions with exact 95% confidence intervals for binomial proportions using the Clopper-Pearson method [37]. Responses with non-overlapping confidence intervals can be considered statistically significantly different from each other.

In order to assess what demographic characteristics were independent predictors of key responses, we developed Poisson regression models using a robust sandwich variance estimator [38]. Each model had a dependent variable of one of five responses that were selected for regression modeling *a priori*, which included concern about welfare, attention to labels, and willingness to pay extra for eggs, chicken breast, or restaurant entrée. The demographics’ characteristics (independent variables) included in models were those expected to be relevant to retailers who want to understand attitudes of a particular customer segments. This included age, household income, gender, and geographic region. The Poisson models were used to generate predicted values of the adjusted probability for having a particular response among each demographic subgroup. Poisson regression was selected because, in practice, these probabilities can be interpreted as the expected proportion (percentage) of each demographic subgroup that would report a particular response while holding other demographic characteristics in the models constant (at their marginal proportions). For simplicity, we refer to these modeled values as *expected proportion* values throughout. Wald tests were used to assess the statistical significance of each independent variable (assessing all levels of the variable simultaneously) [39]. We also tested the interactions between age and income for the three questions on the willingness to pay based on a priori hypotheses that the relationship between income and the willingness to pay may vary by generation.

## 3. Results

### 3.1. Respondent Characteristics

The survey respondents (*N* = 1000) were about half male (45%) with about half (50%) who were less than 45 years old (see Table 1). Almost half of respondents had a college degree (47%) and 51% had a total household income of less than $50,000. The South census region had the largest proportion (36%) of respondents, which is consistent with this region being the most populous. The large majority of respondents reported purchasing meat (76%) or eggs/dairy (77%) more than once per month.

### 3.2. Attitudes towards Farm Animal Welfare and Industry

The large majority of respondents (78%) reported being either somewhat or very concerned about the welfare of animals being raised for food (Table 2) with 35% reporting being somewhat concerned and 42% reporting being very concerned. When we asked about individual farming practices that are considered in welfare certification programs, they were all considered important by more than three fourths of respondents. However, those associated with having more space or less confinement generally received the highest level of importance. A slightly smaller proportion of respondents (76%, 95% CI: 72–80%) reported it was somewhat or very important that farms provide pain control when conducting castration, beak trimming, and other procedures to the animals.

We asked about third-party assessment of welfare conditions in two different ways. In the context of questions on indicators of good welfare, 82% agreed that inspections by outside government or independent entities was an important indicator of welfare. In the context of questions about purchasing decisions, 78% agreed that there should be an objective third party checking on the welfare of farm animals (rather than just the producer itself). There was strong agreement between these two items with 75% of respondents endorsing both. In contrast, only about half of respondents reported having trust in meat, egg, and dairy industries to treat animals well. The dairy industry received the highest level of trust (56% somewhat or completely trust), which was significantly higher than trust in the beef (49%), poultry (46%), or pork industry (45%). The egg industry received the second highest trust level (53%). 

### 3.3. Consumer Purchasing Decisions

What do consumers expect when purchasing ASF in a retail environment? The majority of consumers (70%) report paying attention to labels on ASF that indicate how the animals were raised (Table 3). Overall, 74% report paying more attention to these labels than five years ago and, even among those who pay little attention, 38% still reported paying more attention than five years ago. Consumers appear to see a benefit in having their retail store making stocking decisions related to animal welfare. A total of 70% of respondents agreed that they want their stores to carry a greater variety of welfare certified ASF than they currently offer. In order to confirm that respondents were considering each item individually when rating importance, we also asked this item in a negative form and obtained similar results with only 33% indicating that they don’t care if their store carries products with certified products. When asked about the importance of different types of information, 76% of participants said that knowing the animal was raised without antibiotics was somewhat or very important to their purchasing decision. This was matched almost exactly in importance of knowing the animal did not suffer (75%).

### 3.4. Willingness to Pay for Welfare Certified Products

Almost 70% of respondents reported willingness to pay ≥$0.50 extra for a dozen eggs or for 1pound of chicken breast if the welfare of the chickens were verified under a trustworthy welfare certification (Table 4). Not surprisingly, the proportion willing to pay declined as the price premium increased with 27% willing to pay ≥$1.50 extra and 2% willing to pay ≥$3.50 extra for welfare-certified eggs. Similarly, 25% were willing to pay ≥$1.00 extra and 3% were willing to pay ≥$4.00 extra for welfare-certified chicken breast. The weighted average of the reported amount willing to pay extra (including those not willing to pay extra at all) was $0.79 for eggs (a 32% premium) and $0.96 for 1 pound of chicken breast (a 48% premium). The level of concern for the welfare of farm animals was strongly associated with their willingness to pay. The weighted mean willingness to pay for welfare-certified eggs was $0.33 for those who were not concerned at all, $0.50 for those not too concerned, $0.71 for those somewhat concerned, and $1.09 for those very concerned. The weighted mean willingness to pay for welfare-certified chicken for these same groups was, respectively, $0.41, $0.58. $0.85, and $1.33. We further evaluated the relationship among lower income respondents in order to identify consumers who may feel like they are economically forced to make purchasing choices that conflict with their values. Among respondents with household income <$50,000, 59% saw animal welfare as important but either would not pay extra for welfare-certified chicken or would not pay more than $0.50 extra. When asked to make a decision between paying extra for welfare-certified products as opposed to paying the same but eating smaller portions of meat, eggs, or dairy, respondents were evenly divided with 43% choosing each of these options and another 14% reporting being unsure/unable to answer.

The majority of respondents (83%) reported that they would be likely to choose a restaurant because it serves welfare-certified animal products and says so on the menu and 57% reported that they were both likely to choose such a restaurant and would be willing to pay ≥$5.00 extra per entrée (Table 4). Among those likely to choose such a restaurant, the large majority of respondents reported being willing to travel farther to eat there with 33% willing to travel farther but no more than 10 min extra and 46% willing to travel more than 10 min extra.

### 3.5. Demographic Predictors of Consumer Attitudes and Willingness to Pay

In regression models, gender and age were significant independent predictors of being somewhat or very concerned about farm animal welfare (Table 5). Women were more likely than men to report concern (expected proportion 82% vs. 72%) and respondent <30 years old or 30–45 were the most like to report this outcome (expected proportions 78% and 86%, respectively). Household income and geographic regions were not significantly associated with the level of concern about animal welfare of animals raised for food. Similarly, both women and respondents ≤44 years of age had the highest expected proportion paying attention to labels that indicate how the farm animals were raised. Respondents with household income >$50,000 were also more likely to pay attention to those labels, but the geographic region was not associated with attention to labels.

Decreasing age was a significant independent predictor of willingness to pay extra. For certified eggs, the expected proportion willing to pay extra decreased from 85% for those <30 years old to 55% for those >60 years old. Similarly, for certified chicken, the proportion willing to pay extra decreased from 83% for those <30 years old to 50% for those >60 years old. Even though household income was also significantly associated with willingness to pay for these items (*p* = 0.02 for certified eggs and 0.05 for certified chicken), the differences between income groups was smaller than for age. The two upper income brackets ($50,000–$100,000 and >$100,000) were similar in the expected proportion willing to pay extra (71% to 74%). There were no significant differences in reported willingness to pay by sex or age, but increasing income was a significant independent predictor. Interactions between age and income were assessed for the three questions on willingness to pay, but none were statistically significant (all *p* > 0.20).

## 4. Discussion

Overall, our study identified high levels of reported concern for and interest in the welfare of farm animals as well as reported willingness to pay for higher welfare food products. The large majority of consumers reported being either somewhat or very concerned about the welfare of animals raised for food (42% and 35%, respectively). We also found a trend for younger respondents and female consumers to report increased concern for welfare. McKendree et al., reported a lower overall level of concern for livestock (46%) than we did but a similar association between decreasing age and the level of concern [40]. Many previously published studies reporting consumer concern were specific to a particular industry and tended to also report slightly lower overall levels of concern. For example, 63% of US dairy consumers were concerned with cattle welfare [24]. These differences may be attributable to differences in the population captured in other studies. Wolf’s study respondents were older than in our study with more than half above 55 years of age. Another plausible explanation for the differences is that consumers may conceptualize concern for farm animals in a general sense (as we posed the question) differently than they do for an individual species. Additionally, if the relationship we identified between age and concern is reflective of a generational shift, one would expect older studies to find lower levels of concern. Despite these differences, all studies we are aware of showed a significant level of concern for the welfare of farm animals among US consumers.

Consumer purchasing decisions for ASF can be complex because of the many choices available. A 2015 review reported that consumers sometimes rely on their perceptions of how the food will affect them personally (i.e., the quality or safety of the food) but also consider more altruistic factors such as the perceived impact on the environment or the welfare of the animals raised to create those products [41]. A similar review analyzed several studies that demonstrate a hierarchy of considerations in purchasing decisions in which food safety is typically considered most important—even a requirement—and animal welfare is typically considered only after safety and quality are assured [42]. This dichotomy in decision-making was demonstrated in our respondents’ attitudes to various farming practices. For example, 76% of respondents reported that it was important when making purchasing decisions to know that the animals did not receive antibiotics. There is likely a mix of perceptions on the potential benefits of reducing antibiotics among US consumers. Some consumers may perceive the practice purely as an indicator of safer food while others may perceive that it also has implications for animal welfare. A previous study, for example, found that 47% of consumers believe removing antibiotics indicates a better life for animals. In fact, reducing or eliminating the use of antibiotics at sub-therapeutic levels for growth promotion may be beneficial for public health, but it does not, in itself, convey any benefit for animal welfare. Withholding appropriate antibiotic treatment from farm animals who are ill may even have negative welfare implications [43]. To have a positive impact on farm animal welfare, efforts to reduce antibiotic use would also need to be accompanied by changes in husbandry practices that improve the health of the animals and reduce their risk of infection in the absence of antibiotics. This is a benefit of some welfare certifications that have space and environmental standards that promote animal health and support reduced antibiotic use.

Most consumers, nevertheless, also clearly value higher-welfare farming practices in the absence of a direct benefit to them. When asked about considerations for purchasing decisions, for example, the level of importance in knowing the animal did not suffer (75%) was nearly identical to that for reduced antibiotic use. Similarly, when asked about indicators of good welfare, pain management for surgical procedures and reduced confinement were perceived as important by the large majority of respondents in our study even though these practices are likely not perceived to be related to improved consumer health or product quality. These results are consistent with the Wolf et al., 2016, finding that 68% of US dairy consumers would vote to ban castration without pain control and Dransfield et al.’s finding that European consumers have higher satisfaction with pork that was produced outdoors (vs. indoors) in more natural settings even when they could not taste a difference in the meat [44].

Consumers appear to see a benefit in inspections and certification from either third-parties or the government to ensure the animals’ conditions. When asked about purchasing decisions, most respondents (78%) thought it important to know that animal-welfare assessments are conducted by an independent third party or the federal government (and not just the producer) and 82% saw inspection by outside government or independent entities as an important indicator of animal welfare. These findings are consistent with studies in Europe where consumers reported more confidence in government labels than they did in private brands [45]. Similarly, Wolf, et al. found that dairy consumers perceived that the USDA was the most likely to provide accurate information on dairy cattle welfare. This need for verification of welfare conditions may be related to the weak degree of trust reported (roughly 50%, on average) in the meat, egg, and dairy industries to treat animals well. Trust did vary by industry, however, with the dairy industry garnering modestly, but significantly, higher trust than the beef, poultry, or pork industries. These differences may be related to efforts by the dairy industry to portray themselves as having high welfare conditions or the fact that slaughter is not required to create the dairy products [46].

ASF coming from farms with higher welfare standards would be considered *credence goods* since the consumer cannot typically detect any difference in the product when compared to conventional sources after it is purchased [3,45,47,48]. This lack of intrinsic evidence of welfare conditions along with uncertainty in whether to trust the industry point to the need to have accurate, transparent labeling so that consumers who are willing to pay more for ASF from higher welfare sources know that they are purchasing what they intended. Furthermore, economic studies have demonstrated that fraudulent or misleading claims that cannot be easily verified by the consumer tend to persist over time because perpetuating those claims is profitable for the business and the claims often fail to elicit consumer attention especially when the price is low [48]. In contrast, consumer awareness of inaccurate or misleading welfare label claims that are intended purely for marketing purposes could have the effect of reducing consumer trust in all welfare-related label claims [23].

Because ASF in the US with meaningful welfare certifications tend to be more expensive than products from conventionally raised animals, we wanted to understand consumers’ willingness to pay for these products and what consumer segments would be most likely to pay extra. Using a direct solicitation of willingness to pay discreet amounts, we found that about two-thirds of consumers reported a willingness to pay extra for eggs or chicken breast with a trustworthy welfare certification or for an entrée from a restaurant that serves welfare-certified animal products. This proportion is modestly higher than that found in other studies. For example, a 2005 study found that 57% of European Union consumers were prepared to pay more for eggs from animal welfare-friendly production systems [6]. Our respondents reported, on average, willingness to pay $0.79 extra for eggs and $0.96 extra for 1 pound of chicken breast. Additionally, willingness to pay was strongly associated with the level of concern for farm-animal welfare. In particular, those who were very concerned or somewhat concerned reported a willingness to pay a price premium of 38% and 28%, respectively, for welfare-certified eggs and a premium of 67% and 43%, respectively, for welfare-certified chicken. Other studies that focused on measuring the willingness to pay have typically evaluated a variety of specific rearing conditions for one animal species and used more nuanced methods for assessing the outcome. For example, Norwood et al. employed the calibrated auction-conjoint valuation method and found that US consumers were willing to pay an average of $0.95 (103%) more for a dozen eggs from free-range hens in an aviary and a pasture system when compared to what they would pay for eggs from hens raised in conventional cage systems (base price of $0.92). This same study reported lower marginal willingness to pay ($0.61; 66%) for a dozen eggs from hens raised in a barn and substantially higher marginal willingness to pay ($1.31; 142%) for free-range hens in an aviary and a pasture system who were also raised with organic feed likely because of perceived additional environmental or health benefits associated with organic farming.

We found high levels (57%) of reported willingness to pay ≥$5.00 extra for an entrée from a restaurant that serves welfare-certified animal products. Overall, the proportion willing to pay extra at a restaurant was lower than for eggs and chicken purchases likely because of the extra step of needing to choose the restaurant. Among those would choose such a restaurant, most (88%) reported willingness to travel farther to eat at a restaurant that serves welfare certified animal products. We are not aware of comparable published studies on marginal willingness to pay for restaurant entrées or willingness to travel.

Intuitively, household income might be expected to be strongly associated with willingness to pay. In adjusted regression models, however, we found relatively small differences between income groups in reported willingness to pay with no statistically significant differences between those with household income $50,000–$100,000 and >$100,000 (i.e., all confidence intervals overlapped). This finding would imply that those with even modest income have similar attitudes towards the decision to purchase ASF from higher welfare sources. We found no significant statistical interactions between age and income indicating that the relationship between income and willingness to pay did not differ meaningfully between generations. In general, reported concern for farm-animal welfare was strongly associated with higher willingness to pay. We also identified a large subgroup of those with household income <$50,000 who were concerned about farm-animal welfare but didn’t think they would pay more than $0.50 extra for welfare certified products. These consumers are, therefore, positioned to make choices that conflict with their values. As less expensive high-welfare options become available, some of these consumers might be expected to change their purchasing decision away from conventional ASF. Even though women reported higher levels of concern for farm animal welfare, we found that men and women were similar in terms of their reported willingness to pay for higher welfare products. This may suggest that men and women may ultimately make similar purchasing decisions for higher welfare ASF even if, on average, they tend towards different motivations. Availability of higher welfare products varies by geographic region, but, in our study, consumer interest in and willingness to pay do not vary meaningfully by the census region. This would imply that, when making stocking decisions for higher welfare products, retailers should not make assumptions about consumer interest based on the store’s region. Our findings are generally consistent with a meta-analysis conducted by Lagerkvist et al., which found that age and income but not geographic region effect willingness to pay for higher welfare products [49]. When asked to make a decision between paying more for ASF from animals that were raised more humanely when compared to consuming less at the same total cost, responses were mixed. This indicates that these types of decisions may be difficult and that consumers may take varying approaches to the issue of increased cost for higher welfare items.

Assessing consumer demand for new products is difficult and consumer decisions are particularly difficult to study when animal welfare issues are involved [50]. As with other studies, there may be a gap between our respondents’ reported interest in purchasing certain products and their actual purchasing decisions [51]. In particular, respondents may have over-reported positive responses such as their level of concern or willingness to pay due to social desirability bias. The perception that concern about the welfare of farm animals would be viewed favorably by others could influence some respondents to over-report those items. Respondents who were reluctant to provide truthful answers that could be viewed negatively may also have chosen not to provide an answer, but the proportion of respondents who did not provide an answer was relatively small and ranged from 1% on the question about concern for farm animal welfare up to 10% for the questions on willingness to pay extra at a restaurant. We attempted to reduce this type of bias by describing the goal of the survey in neutral terms, mixing in both positive and negative items, and by ensuring that respondents were blinded to the study funding source. Our data on willingness to pay is also subject to two limitations. First, we collected WTP by providing discreet choices when actual WTP may have been between the choices given, which potentially reduces the precision of our measures. As with almost any assessment of consumer decision-making, some participants may respond differently to hypothetical scenarios than they would when facing real purchases (*hypothetical bias*). A study by Lusk et al., however, found that marginal willingness to pay for meat with varying quality attributes did not differ significantly between hypothetical and non-hypothetical testing.

## 5. Conclusions

In conclusion, our findings suggest that many US consumers across all demographics may be willing to seek out and pay more for higher welfare animal products both in supermarkets and in restaurants provided that claims made about animal treatment are trustworthy, e.g., supported by an independent party. As mentioned in the introduction, three certification systems that include auditing of farm conditions are being adopted in the US and can be used for distinguishing conventional ASF from those coming from animals raised under higher welfare conditions. In light of high levels of consumer concern around farm animal welfare, greater awareness toward labels, and low levels of trust in the animal agriculture industries, retailers interested in capturing this potentially lucrative market for higher welfare products should understand which certifications and claims meaningfully and verifiably improve farm animals’ welfare.

## Figures and Tables

**Table 1:** Characteristics of Respondents (*N* = 1000).

Characteristic	%
Sex	
Male	45
Female	55
Age, year	
Under 30	23
30–44	27
45–59	26
60 & over	25
Education	
High school or less	28
Some college	25
College graduate/post-graduate	47
Total Household Income	
Below $50,000	51
$50,000–$99,999	33
Above $100,000	13
Can’t Answer	3
US Census Region	
Northeast	20
Midwest	23
South	36
West	21

Proportions calculated using survey weights.

**Table 2:** Attitudes towards farm conditions and the meat, egg, and dairy industry.

Item	%	95% CI
Concern about the welfare of animals that are raised as food for people to eat. *% somewhat or very concerned*	78	75–81
Believe that there should be an objective third party checking on the welfare of animals on farms rather than just the company itself. *% agree or strongly agree*	78	75–81
Importance of practices for indicating good treatment of farm animals. *% somewhat or very important*.		
Farms raise animals with shelter, resting areas, and sufficient space	84	80–87
Farms do not confine animals so tightly that they can barely move	84	80–87
Farms are inspected by outside government or independent entities to verify that they are treating animals well	82	78–86
Farms provide healthy enough living conditions so that the animals do not need to be routinely fed antibiotics to prevent illness in the animals	78	74–82
Animals spend most of their time outdoors on pasture	77	73–81
Animals have the ability to engage in natural behaviors since they would live in natural conditions	77	73–81
Farms do not confine animals in cages where they can’t turn around or extend their limbs	76	72–80
Farms provide pain control when conducting castration, beak trimming, or other procedures	76	72–80
Trust in industry to treat the animals they raise for food well. *% somewhat or completely trust*		
Dairy	56	53–59
Egg	53	49–56
Beef	49	46–52
Poultry	46	43–49
Pork	45	42–48

Results calculated using survey weights. CI: Confidence Interval.

**Table 3:** Consumers’ considerations when making purchasing decisions.

Item	%	95% CI
Pay attention to labels on meat, eggs, and dairy products saying how the animal was raised. *% some or a lot of attention*	70	66–73
Do not care if store carries products with certifications that ensure that farm animals are treated well. *% agree or strongly agree*	33	30–36
Would like stores to carry a greater variety of welfare-certified meat, eggs, and dairy products than they currently offer. *% agree or strongly agree*	75	72–78
Importance of information when making a purchasing decision. *% somewhat or very important*		
Knowing the animal did not receive antibiotics	76	72–80
Knowing the animal did not suffer when it was raised on the farm	75	71–79
Knowing the animal was treated well	74	70–78
Knowing the product is labeled as natural	69	65–74
Knowing the product is labeled as USDA Organic	68	64–73
Where the animal was raised—indoors or outdoors	65	61–69

**Table 4:** Consumer willingness to pay for welfare-certified meat, eggs, and dairy.

Item	% *	95% CI
Willingness to Pay for a Dozen Eggs that Came from Hens Whose Welfare was Verified under a Trustworthy Welfare Certification *		
Would not pay extra	31	28–34
$0.50 extra	41	38–45
$1.50 extra	16	13–18
$2.50 extra	9	7–11
≥$3.50 extra	2	1–3
**Willingness to Pay for 1 pound of Chicken Breast that Came from Hens Whose Welfare was Verified under a Trustworthy Welfare Certification**		
Would not pay extra	30	27–33
$1.00 extra	37	34–40
$2.00 extra	15	13–18
$3.00 extra	7	6–9
≥$4.00 extra	3	
**Willingness to Pay for Entree at a Restaurant that Serves Welfare-Certified Animal Products**		
Would not pay extra **	43	40–47
<$5.00 extra	41	37–44
$5.00–$10.00 extra	13	11–15
>$10.00 extra	3	2–4

* Among those who provided an answer. ** Includes those unlikely to choose a restaurant based on serving welfare certified products.

**Table 5:** Modeled probabilities of five outcomes related to attitudes toward farm animal welfare and reported willingness to pay for higher welfare products by four key demographic characteristics.

Demographic Predictors	Model 1: Concern About Welfare ^a^		Model 2: Attention to Labels ^b^		Model 3: Willing to Pay More, Certified Eggs ^c^		Model 4: Willing to Pay More, Certified Chicken ^c^		Model 5: Willing to Pay More, Restaurant Entrée ^d^	
	Prob (95% CI)	*p-*Value	Prob (95% CI)	*p*-Value	Prob (95% CI)	*p-*Value	Prob (95% CI)	*p-*Value	Prob (95% CI)	*p-*Value
Age, year										
<30	78 (72–84)		76 (70–82)		85 (80–91)		83 (78–89)		68 (61–75)	
30–44	86 (82–91)	<0.001	77 (72–82)	<0.001	74 (68–80)	<0.001	75 (69–80)	<0.001	59 (51–66)	0.11
45–59	75 (69–81)		64 (58–70)		63 (57–70)		63 (56–70)		56 (49–63)	
≥60	70 (64–76)		58 (52–64)		55 (48–62)		50 (43–57)		40 (33–48)	
Annual household income, $										
<50,000	76 (72–80)		65 (61–70)		65 (60–69f		63 (59–68)		52 (47–57)	
50,000-100,000	79 (74–83)	0.67	73 (68–78)	0.05	73 (68–78)	0.02	71 (66–76)	0.05	58 (52–64)	0.02
>100,000	78 (71–85)		72 (64–80)		74 (66–81)		73 (65–80)		61 (52–69)	
Gender										
Female	82 (79–86)		65 (60–69)		69 (64–73)		66 (61–70)		52 (47–57)	
Male	72 (67–76)	<0.001	72 (68–76)	0.02	69 (64–73)	0.96	68 (64–73)	0.44	58 (53–63)	0.11
US Census Region										
Midwest	78 (72–85)		66 (59–73)		69 (62–75)		68 (61–75)		53 (44–61)	
Northeast	77 (72–83)	0.64	66 (60–73)	0.36	68 (62–75)	0.99	66 (59–72)	0.69	58 (50–66)	0.74
South	79 (74–83)		72 (67–77)		69 (64–74)		69 (64–74)		56 (50–62)	
West	74 (68–80)		68 (62–74)		68 (62–74)		64 (58–71)		53 (46–60)	

All results are from a generalized linear model with Poisson distribution using robust variance estimate accounting for survey weights and adjusting for all four variables in the model and among respondents for whom all relevant data was available. Prob: Modeled probability (expected proportion) reporting each response. ^a^: Somewhat or very concerned about the welfare of animals that are raised as food for people to eat. ^b^: Pay some or a lot of attention to labels on meat, eggs, and dairy products saying how the animal was raised. ^c^: Willing to pay ≥$0.50 extra for a dozen eggs or 1 pound of chicken breast that came from hens whose welfare was verified under a trustworthy welfare certification. ^d^: Likely to choose a restaurant that serves welfare-certified animal products and are willing to pay ≥$5.00 extra for an entrée from that restaurant.

## Supplementary Materials

The following are available online at http://www.mdpi.com/2076-2615/8/8/128/s1, File1: Content of Online Questionnaire.
