# Supplementary material for: Are They Buying It? United States Consumers’ Changing Attitudes toward More Humanely Raised Meat, Eggs, and Dairy
Source: Animals (Basel). 2018 Jul 25;8(8):128. doi: 10.3390/ani8080128 (PMC6116027; doi:10.3390/ani8080128)
Supplement: Supplementary file 1 [file animals-08-00128-s001.pdf]

Category	Questions
**Respondent characteristics**	1. What was your age on your last birthday?<br>2. Please indicate your gender. *Male, Female*<br>3. In what state do you live?<br>4. What is your race? *White/Caucasian, Black/African American, Latino/Hispanic, Asian/Pacific Islander, Native American, Other race, Mixed Race*<br>5. What is the last year of schooling that you have completed? *1–11th grade, High school graduate, Some college, College graduate, Post-graduate school*<br>6. In which of the following ranges, does your total annual HOUSEHOLD income fall, before taxes? *Below $30,000, Between $30,000 and $49,999, Between $50,000 and $74,999, Between $75,000 and $99,999, Between $100,000 and $149,999, $150,000 or more, Can't answer*<br>7. Are you the primary grocery shopper in your household, or is there someone else who does most of the grocery shopping for you and your family? *Primary grocery shopper, share equal responsibility as primary grocery shopper, am not responsible for grocery shopping, Can't answer*<br>8. Please tell us about some shopping activities and how often, if at all, you engage in them. For the following, please tell us how often you do each. *A few times a week or more, About once every week or two, About once a month, Rarely, Never, Can't answer*<br>• Purchase meat products for yourself or your family, such as chicken, beef, turkey, or pork products<br>• Purchase eggs or dairy products for yourself or your family
**Attitudes towards farm conditions and the meat, egg, and dairy industries**	9. Generally speaking, how concerned are you about the welfare of animals that are raised as food for people to eat? *Very concerned, Somewhat concerned, Not too concerned, Not concerned at all, Can't answer*<br>10. Please rate these practices on how important you think they are for indicating good treatment of farm animals. *on an 11-point scale from not important at all to extremely important*<br>(Order randomized)<br>• Farms do not confine animals in cages where they can't turn around or extend their limbs<br>• Farms do not confine animals so tightly that they can barely move.<br>• Farms provide healthy enough living conditions so that the animals do not need to be routinely fed antibiotics to prevent illness in the animals.<br>• Farms provide pain control when conducting castration, beak trimming or other procedures.<br>• Animals spend most of their time outdoors, on pasture.<br>• Farms raise animals with shelter, resting areas, and sufficient space.<br>• Animals have the ability to engage in natural behaviors as they would in natural conditions.<br>• Farms are inspected by outside government or independent entities to verify that they are treating animals well<br>11. Below are some industries that produce food. How much trust do you have that each of following industries treat the animals they raise for food well? *on an 11-point scale from do not trust at all to completely trust*<br>(Order randomized)<br>• Beef industry<br>• Pork industry<br>• Egg industry<br>• Poultry industry<br>• Dairy industry
**Considerations when making purchasing decisions**	12. Often times, food labels for things like meat, eggs, and dairy products, will say how the animal was raised. When making decisions about what to buy, how much attention do you give to these labels? *A lot of attention, Some attention, Not too much attention, No attention, Can't answer*<br>13. Generally speaking, are you paying more attention to these kinds of labels than 5 years ago? *Yes, No, Can't answer*

	14. How would you rate each statement? *on an 11-point scale from strongly disagree to strongly agree*<br>(Order randomized)<br>• I do not care if my store carries products with certifications that ensure that farm animals are treated well<br>• I would like my stores to carry a greater variety of welfare certified meat, eggs, and dairy products than they currently offer<br>• I think there should be an objective third party checking on the welfare of animals on farms rather than just the company itself<br>15. Below are some things that some people consider when they purchase meat, eggs, and dairy products. Regardless of how often you consume meat, eggs, and dairy products, for each of the following, please indicate how important that particular factor is to you when making purchasing decisions. *11-point scale from not important at all to extremely important*<br>(Order randomized)<br>• Knowing the animal did not receive antibiotics<br>• Knowing the product is labeled as USDA Organic<br>• Knowing the product is labeled as natural<br>• Where the animal was raised—indoors or outdoors<br>• Knowing the animal did not suffer when it was raised on the farm<br>• Knowing the animal was treated well
**Willingness to pay or travel extra for higher-welfare goods**	16. If, on average, a dozen eggs cost $2.50, how much would you be willing to pay for a dozen eggs that came from hens whose welfare was verified under a trustworthy welfare certification? *Would not pay anything extra, $3.00 per dozen, $4.00 per dozen, $5.00 per dozen, $6.00 per dozen, More than $6.00 per dozen, Can't answer*<br>17. If, on average, chicken breasts cost $2.00 per pound, how much would you be willing to pay for chicken that came from chickens whose welfare was verified under a trustworthy welfare certification? *Would not pay anything extra, $3.00 per pound, $4.00 per pound, $5.00 per pound, $6.00 per pound, More than $6.00 per pound, Can't answer*<br>18. And if you had to choose, would you rather spend more money for meat, eggs, and dairy products from animals that were raised more humanely spend the same amount as you spend now, but for a smaller portion of meat, eggs, and dairy products from animals that were raised more humanely? *Pay more, Pay the same, but less meat, eggs or dairy, Can't answer*<br>19. How likely would you be to choose a restaurant because it serves welfare certified animal products and says so on the menu? *Very likely, Somewhat likely, Somewhat unlikely, Very unlikely, Can't answer*<br>20. How much more would you be willing to pay for an entree at a restaurant that serves welfare certified animal products? *Would not pay any more for welfare certified, Up to $5.00 more, Between $5.00 and $10.00, Over $10.00 more, Can't answer*<br>21. How much more would you be willing to travel to a restaurant that serves welfare certified animal products? *Would not travel further for welfare certified, 10 min, 10 to 20 min, Over 20 min, Can't answer*
